# Maternal Microbiome in Gestational Diabetes Mellitus: Mechanisms, Biomarkers, and Therapeutic Perspectives

**DOI:** 10.3390/life16071065

**Published:** 2026-06-26

**Authors:** Diana-Maria Deaconu, Gratiela Gradisteanu Pircalabioru, Octavian Savu

**Affiliations:** 1 Faculty of Biology, Splaiul Independenti 91-95010041, University of Bucharest, 050095 Bucharest, Romania; d.deaconu20@s.bio.unibuc.ro; 2 eBio-Hub Centre of Excellence in Bioengineering, National University of Science and Technology Politehnica Bucharest, 060042 Bucharest, Romania; 3 Academy of Romanian Scientists, Ilfov Street, No. 3, 050044 Bucharest, Romania; 4 “N.C. Paulescu” National Institute of Diabetes, Nutrition and Metabolic Diseases, 020042 Bucharest, Romania; octavian.savu@umfcd.ro; 5 Department of Doctoral School, “Carol Davila” University of Medicine and Pharmacy, 5th District, 050474 Bucharest, Romania

**Keywords:** gestational diabetes mellitus, maternal microbiome, dysbiosis, insulin resistance, metabolic endotoxemia, biomarkers, probiotics

## Abstract

Gestational diabetes mellitus (GDM) is an increasingly prevalent metabolic disorder of pregnancy, driven by rising maternal age, obesity, and complex metabolic–inflammatory interactions. Emerging evidence implicates the maternal microbiome as a key modulator of metabolic adaptation during gestation; however, its precise role in GDM pathogenesis remains incompletely defined. This narrative review synthesizes current knowledge on microbiome alterations across gut, vaginal, and oral niches, focusing on their contribution to insulin resistance, metabolic endotoxemia, and immune dysregulation. GDM is consistently associated with reduced microbial diversity, depletion of beneficial taxa (e.g., *Akkermansia*, *Bifidobacterium*, *Faecalibacterium*), and expansion of pro-inflammatory pathobionts, which collectively may impair intestinal barrier integrity and promote low-grade systemic inflammation. These mechanisms are linked to altered insulin signaling and adverse maternal–fetal outcomes. In parallel, microbiome-derived metabolites and early taxonomic signatures have been proposed as potential biomarkers for first-trimester risk stratification, offering an opportunity to overcome the limitations of late diagnostic approaches such as the oral glucose tolerance test. Despite these advances, most available evidence remains associative, with substantial heterogeneity across studies and limited mechanistic validation. The clinical utility of microbiome-based interventions—including dietary modulation, prebiotics, and probiotics—remains promising but inconclusive, with outcomes highly dependent on individual, microbial, and methodological factors. Overall, the maternal microbiome represents a compelling but still evolving target in GDM research. Future progress will depend on standardized methodologies, longitudinal multi-omics studies, and the development of precision medicine approaches capable of integrating microbial, metabolic, and host data. Such advances may enable earlier diagnosis, targeted prevention, and ultimately the disruption of intergenerational metabolic risk.

## 1. Introduction

Gestational diabetes mellitus (GDM) represents one of the most common metabolic complications occurring during pregnancy, characterized by glucose intolerance with onset or first recognition during the second or third trimester of gestation [1]. Globally, the incidence of GDM has seen a significant escalation, aligning with the secular trends of maternal adiposity, sedentary lifestyles, and shifting demographic patterns toward advanced maternal age [2]. The clinical spectrum of GDM extends beyond transient maternal hyperglycemia; it poses immediate risks of obstetric complications, including preeclampsia, macrosomia, operative delivery, and neonatal hypoglycemia [3]. Long-term epidemiological tracking reveals that women diagnosed with GDM face an approximately ten-fold higher relative risk of developing type 2 diabetes mellitus (T2DM) postpartum, while their offspring exhibit enhanced susceptibility to juvenile obesity, early-onset metabolic syndrome, and cardiovascular dysfunction [4]. Consequently, GDM is no longer viewed as an isolated gestational anomaly but as a critical window that modulates lifelong cardiometabolic trajectories across generations.

### 1.1. Scope and Type of Review

This article represents a narrative review that aims to synthesize and critically interpret current evidence regarding the role of the maternal microbiome in the pathogenesis of GDM. The scope of this review encompasses microbial alterations across multiple maternal niches (gut, vaginal, and oral), their mechanistic contribution to metabolic inflammation and insulin resistance, and their impact on maternal–fetal interactions and long-term metabolic programming. In addition, the review explores emerging microbiome-based biomarkers for early detection and discusses potential therapeutic strategies, including microbiome modulation through diet and probiotics. Rather than applying a systematic methodology, this narrative approach allows for an integrative and conceptual analysis of recent advances, highlighting mechanistic insights and identifying knowledge gaps to guide future research.

### 1.2. Literature Search Strategy

Although this manuscript is presented as a narrative review, a structured literature search was conducted across the PubMed, Scopus, and Web of Science databases to identify relevant studies published up to early 2026. The search strategy utilized combinations of MeSH terms and keywords, including: “maternal microbiome”, “gestational diabetes mellitus”, “GDM”, “dysbiosis”, “metagenomics”, and “microbial biomarkers”. Inclusion criteria focused primarily on original peer-reviewed research projects, clinical trials, and observational cohorts investigating microbial composition across multiple maternal niches (gut, vaginal, oral, placental) and neonatal matrices, prioritizing high-quality data published within the last five years to ensure contemporary clinical relevance. Priority was given to human observational studies, longitudinal cohorts, clinical trials, and mechanistic studies with translational relevance. Animal and in vitro studies were included primarily to support mechanistic hypotheses when human evidence remained limited. Preference was given to studies published in English within the last 5–10 years, although seminal earlier publications were also included where relevant.

### 1.3. Emerging Concept: The Microbiome as a Metabolic Organ

The human intestinal tract harbors a complex ecosystem of trillions of microorganisms, collectively referred to as the gut microbiota, which contributes over 3.3 million unique bacterial genes to the host metagenome [5]. Over the past two decades, this microbial community has been redefined from a passive digestive population to a highly reactive, bioreactive metabolic organ functioning at the intersection of host immunity, endocrinology, neurologics, and energy balance [6]. The gut microbiota expands the host’s metabolic repertoire by fermenting otherwise indigestible dietary glycans and modifying endogenous substrates, transforming them into bioactive low-molecular-weight ligands and systemic signaling molecules [7].

As schematically mapped in the integrated neuro–endocrine–metabolic framework, the structural functional landscape of this microbial organ interfaces directly with the host through a multi-layered intestinal architecture. This barrier comprises an outer lumen, a specialized mucus layer, and an organized layer of intestinal epithelial cells sealed by tight junction protein complexes [8]. Within this microenvironment, commensal taxons metabolize complex carbohydrates and dietary fiber into short-chain fatty acids (SCFAs)—primarily acetate, propionate, and butyrate—which act as critical energetic substrates and signaling molecules [9]. These SCFAs are sensed directly at the epithelial interface by targeting specific host G-protein coupled receptors, namely free fatty acid receptor 2 (FFA2/GPR43) and free fatty acid receptor 3 (FFA3/GPR41), expressed on both enteroendocrine cells and resident immune populations [10]. Concurrently, the metabolic organ processes primary bile acids via bacterial bile acid hydrolases (BAHs), converting them into secondary bile acid profiles that engage key metabolic nuclear receptors, specifically the farnesoid X receptor (FXR) and the membrane-bound Takeda G-protein-coupled receptor 5 (TGR5), which orchestrate systemic lipid and glucose clearance [11].

The operational reach of the microbiome as a metabolic organ extends far beyond the local gastrointestinal tract, utilizing distinct neuroendocrine and neuroimmune circuitries to maintain systemic homeostasis. A primary bidirectional pathway is the gut–brain-endocrine axis, which bridges the enteric nervous system (ENS) and local afferent projections of the vagus nerve directly to the central nervous system [12]. Luminal microbial metabolites and local neurochemical signals activate ENS plexuses and vagal afferents, transmitting metabolic status updates to the hypothalamus [13]. This central integration controls the feedback loop of the Hypothalamic–Pituitary–Adrenal (HPA) axis, modulating the systemic release of adrenocorticotropic hormone (ACTH) from the anterior pituitary gland, which subsequently dictates the rhythmic secretion of cortisol from the adrenal cortex [14].

In parallel, this complex organ exercises strict control over host systemic defense and energetic allocation through direct interaction with the host immune cells situated within the lamina propria [15]. In a state of homeostatic balance (eubiosis), the continuous, low-threshold presentation of bacterial ligands and immunomodulatory metabolites trains dendritic cells and macrophages, keeping the secretion of pro-inflammatory cytokines under metabolic control [16]. Through these coupled pathways—vagal transit, HPA axis-driven cortisol dynamics, and peripheral cytokine balancing—the microbiome interfaces directly with distant peripheral organs, participating in the regulation of hepatic gluconeogenesis, skeletal muscle lipid oxidation, and adipose tissue browning [17]. During pregnancy, the maternal gut microbiome undergoes structural and functional modifications, acting as a master endocrine coordinator that assists the maternal host in shifting nutrient partitioning toward the fetal compartment, thereby serving as a critical environmental modifier of maternal metabolic adaptations [18].

### 1.4. Hypothesis: Microbiome Dysbiosis Contributes to Metabolic Inflammation and Insulin Resistance

The foundational hypothesis of this framework posits that a disruption in the structural composition and functional capacity of the maternal gut microbiota—termed dysbiosis—acts as a primary upstream driver of pathological insulin resistance and systemic metabolic inflammation (*metaflammation*) in GDM [19]. Rather than being a secondary consequence of maternal hyperglycemia, dysbiosis disrupts the highly regulated dialog between the intestinal lumen and the host vascular compartment. When the symbiotic microbial profile shifts toward an enrichment of pathobionts, the integrity of the multi-layered intestinal barrier is structurally compromised [20]. This breakdown permits the systemic translocation of microbial structural components, most notably lipopolysaccharide (LPS) derived from the outer membranes of Gram-negative bacteria, directly into the portal and systemic circulation, a state defined as metabolic endotoxemia [21]. Once in circulation, LPS binds to the pattern recognition receptor Toll-like receptor 4 (TLR4) complexed with CD14 on tissue macrophages, dendritic cells, and adipocytes [22]. This binding triggers an intracellular signaling cascade mediated by the myeloid differentiation primary response 88 (MyD88) pathway, which culminates in the activation of nuclear factor kappa B (NF-κB) and c-Jun N-terminal kinase (JNK) [23]. The activation of these pro-inflammatory kinase pathways leads to the aberrant serine phosphorylation of insulin receptor substrate 1 (IRS-1) at positions such as Ser307, directly blocking the canonical tyrosine phosphorylation required for downstream phosphatidylinositol 3-kinase (PI3K) and protein kinase B (Akt) activation. Consequently, the insulin-stimulated translocation of glucose transporter 4 (GLUT4) vesicles to the plasma membrane in skeletal muscle and adipose tissue is profoundly inhibited, precipitating host-wide insulin desensitization [24].

### 1.5. The Role of Metabolite Deficiencies

Beyond the active induction of inflammatory signaling by bacterial endotoxins, the pathogenesis of GDM is accelerated by the critical depletion of protective, homeostatic microbial metabolites [25]. In a state of intestinal eubiosis, commensal saccharolytic bacteria process complex, otherwise indigestible dietary fibers to synthesize high concentrations of short-chain fatty acids (SCFAs), predominantly acetate, propionate, and butyrate. Butyrate serves as the primary obligate energetic substrate for colonocytes, inducing the expression of tight junction proteins—specifically occludin, claudin-1, and zonula occludens-1 (ZO-1)—via the inhibition of histone deacetylases (HDACs) [26]. Propionate and acetate travel via the portal vein to engage free fatty acid receptors 2 and 3 (FFA2/GPR43 and FFA3/GPR41) expressed on enteroendocrine L-cells. This interaction triggers the intracellular exocytosis of glucagon-like peptide-1 (GLP-1) and peptide YY (PYY), which optimize pancreatic insulin exocytosis and maintain the physiological ileal brake [27]. In pregnancies complicated by GDM, a systemic reduction in SCFA-producing taxons (such as *Faecalibacterium prausnitzii*, *Roseburia*, and *Eubacterium*) causes an acute localized and systemic metabolite deficiency. This deficiency strips the enteroendocrine cells of their natural secretagogue triggers, downregulating GLP-1 and PYY secretion, which directly impairs pancreatic beta-cell compensatory insulin output during late gestation [27]. Concurrently, deficiencies in microbial bile acid processing alter the secondary bile acid pool, limiting the activation of the Takeda G-protein-coupled receptor 5 (TGR5) and the farnesoid X receptor (FXR), both of which are required to maintain host lipid clearance and mitochondrial energy expenditure [11].

### 1.6. Host Risk Factors as Drivers of Pre-Gestational Dysbiosis

Traditional risk factors for gestational diabetes, including maternal obesity, advanced age, and genetic background, are increasingly recognized as primary architects of a vulnerable pre-gestational microbiome. Pre-gestational adiposity establishes a baseline enterotype characterized by reduced alpha diversity and an elevated *Firmicutes*-to-*Bacteroidetes* ratio, creating a chronically hyper-endotoxemic luminal environment that primes the maternal host for severe metabolic failure under the physiological stress of pregnancy [28]. Similarly, advanced maternal age drives an age-related loss of highly specialized, anti-inflammatory taxons and a reciprocal expansion of opportunistic pathobionts, diminishing the mucosal capacity to resolve gestational metaflammation [29]. Furthermore, host genetic determinants and ethnic-specific dietary traditions fundamentally shape the intestinal microenvironment, dictating baseline microbial signatures that amplify GDM susceptibility when interacting with modern metabolic stressors [30].

### 1.7. Limitations of Current Screening Methods and the Shift to Early Prediction

Although the conventional oral glucose tolerance test (OGTT) remains the clinical gold standard for diagnosing gestational diabetes mellitus (GDM), its standard deployment in the late second trimester represents a diagnostic delay that misses the critical window for early preventive intervention, thereby underscoring the urgent need for novel, non-invasive first-trimester biomarkers [31].

### 1.8. The Necessity for Early Biomarkers and Mechanistic Perspectives

The current lag between the pathophysiological onset of insulin resistance and the moment of clinical diagnosis (weeks 24–28) underscores a critical need for new assessment tools. Identifying robust biomarkers in the first trimester (T1) and understanding the molecular mechanisms through which the microbiome influences the host are essential pillars for reducing GDM prevalence.

#### 1.8.1. The First Trimester Intervention Window (T1)

To shift the clinical paradigm from reactive management to proactive prevention, diagnostic strategies must pivot toward the first trimester of pregnancy (T1) [32]. During T1, the maternal metabolic landscape is still relatively stable, and the structural integrity of the intestinal barrier has not yet succumbed to the profound physiological changes of late gestation [33]. Identifying early predictive signatures during this early window provides a unique clinical opportunity. Implementing dietary, prebiotic, or target-specific probiotic interventions during T1 allows clinicians to stabilize the maternal gut architecture, optimize SCFA production, and preemptively curb the development of systemic metaflammation well before the physiological onset of severe placental-mediated insulin desensitization [32,34].

#### 1.8.2. Multi-Omic Biomarkers: Beyond Glycemia

Developing high-precision early screening tools requires a shift beyond simple glycemic measurements toward integrated, non-invasive multi-omic biomarker panels [35]. By coupling high-throughput 16S rRNA or shotgun metagenomic sequencing of the maternal stool with liquid chromatography-mass spectrometry (LC-MS) metabolomics, clinicians can map early functional deviations [36]. Prospective cohort profiling reveals that early elevations in circulating branched-chain amino acids (BCAAs—leucine, isoleucine, and valine) and trimethylamine N-oxide (TMAO), paired with an acute drop in circular acetate and butyrate levels, serve as sensitive predictors of downstream GDM development [7,37]. Integrating these multi-omic metrics via machine-learning algorithms enables the construction of personalized risk matrices with predictive capabilities that vastly outperform traditional clinical risk factors [35]. Recent work by Popova et al. demonstrated that incorporation of gut microbiota features into machine-learning models improved prediction of postprandial glycemic responses in women with diet-treated GDM, supporting the concept of microbiome-informed precision nutrition and individualized metabolic risk assessment [37].

#### 1.8.3. The Need for Mechanistic Clarity

Despite the rapid accumulation of descriptive microbiome sequencing data demonstrating structural correlations between dysbiosis and GDM, the field faces a critical need for rigorous mechanistic clarity [38]. The vast majority of human clinical trials remain fundamentally associative, failing to distinguish whether specific microbial shifts are active causal drivers of metabolic disease or merely secondary consequences of the changing maternal hormonal and glycemic environment [38]. Establishing true causality requires a concerted shift toward experimental validation systems. This includes the utilization of germ-free (GF) animal models subjected to humanized fecal microbiota transplantation (FMT) from highly characterized GDM donors, paired with advanced anaerobic multi-organ gut-on-a-chip technologies [39]. Only by isolating individual bacterial strains, identifying their precise biosynthetic gene clusters (BGCs), and characterizing their specific ligand–receptor interactions can researchers confidently design targeted, mechanism-driven therapeutics [37].

#### 1.8.4. Public Health Impact

Elucidating the precise operational pathways of the maternal microbiome carries profound, far-reaching public health implications [40]. GDM does not function merely as a transient complication of pregnancy; it operates as a major vector for the intergenerational transmission of metabolic syndrome, obesity, and type 2 diabetes [4]. By successfully deploying scalable, microbiome-based early screening and therapeutic stabilization strategies at a population level, public health frameworks can effectively intercept this pathological cycle [40]. Optimizing the maternal microbial reservoir not only protects the immediate metabolic health of the mother but also ensures the vertical transmission of a healthy, protective founder microbiome to the neonate, mitigating the global longitudinal burden of non-communicable metabolic diseases for generations to come [41,42].

## 2. Dynamics of the Gut Microbiome Across Trimesters

The maternal gut microbiome is not a static entity; it undergoes profound remodeling, functioning as a “metabolic adapter” that adjusts energy extraction and insulin sensitivity according to the gestational stage Recent syntheses of human and experimental evidence further support the concept that pregnancy is characterized by progressive microbiome remodeling, which may become exaggerated in women who develop GDM [43].

### 2.1. Trimester-Specific Shifts in the Maternal Gut Microbiota

During the first trimester (T1) of a normal pregnancy, the maternal gut microbiome exhibits a structural configuration that closely resembles a non-pregnant adult eubiotic ecosystem. This phase is characterized by elevated alpha diversity and a stable core composition dominated by the phyla *Bacteroidetes* and *Firmicutes*. Highly specialized saccharolytic taxons, such as *Faecalibacterium prausnitzii* and members of the *Ruminococcaceae* family, thrive within this environment, continuously producing high levels of short-chain fatty acids (SCFAs) like butyrate and propionate [43,44]. These metabolites reinforce epithelial tight junction protein complexes, maintaining low baseline intestinal permeability and a homeostatic, regulatory T-cell-dominated immune tone within the lamina propria.

As pregnancy transitions into the second trimester (T2) and reaches the third trimester (T3), the continuous accumulation of placental hormones exerts profound selective pressures on the maternal intestinal niche. By T3, the gut microbiota undergoes a dramatic, systemic reorganization often described as an adaptive “physiological dysbiosis”. This late-gestational phase features a significant contraction of alpha diversity, a marked expansion of the phylum *Proteobacteria*, and a reciprocal decline in strict anaerobic SCFA producers [43,44].

From a functional metagenomic perspective, the T3 microbiome shifts toward an upregulation of pathways dedicated to lipopolysaccharide (LPS) biosynthesis and highly efficient carbohydrate harvesting. Fecal microbiota transplantation (FMT) experiments from healthy T3 human donors into germ-free mice successfully replicate this metabolic phenotype, actively inducing increased adiposity, insulin desensitization, and elevated inflammatory cytokine profiles in the recipients [38]. This underscores that trimester-associated microbial remodeling functions as an evolutionary adaptation designed to deliberately induce transient maternal insulin resistance, thereby maximizing nutrient availability to support rapid late-stage fetal growth.

### 2.2. The Physiological Stabilization of the Vaginal Microbiome

In striking contrast to the high structural complexity and shifting trimesteral dynamics of the gastrointestinal tract, the healthy maternal vaginal microbiome undergoes a process of profound simplification. Across gestation, this niche transitions into a highly stable state characterized by exceptionally low alpha diversity and the absolute dominance of the genus *Lactobacillus*—specifically *L. crispatus*, *L. jensenii*, *L. gasseri*, and *L. iners* [45,46].

Driven by surging systemic estrogen levels, the vaginal epithelium proliferates and stores vast reserves of intracellular glycogen. Upon desquamation, this glycogen is catabolized into simpler oligosaccharides by host alpha-amylases, providing an exclusive nutrient source for resident lactic acid-producing bacteria [45,47]. The subsequent fermentation of these substrates generates high concentrations of lactic acid, dropping the local vaginal pH to a highly acidic range.

This extreme acidity, paired with the local secretion of hydrogen peroxide and bacteriocins, creates a hostile biochemical shield that aggressively prevents the colonization or ascent of opportunistic uropathogens and dysbiotic pathobionts [45]. The primary evolutionary purpose of this hyper-stable, low-diversity vaginal state is to secure a protective microbial pipeline for parturition. As the neonate transits the birth canal during vaginal delivery, it is saturated by this maternal vaginal inoculum, providing critical founder taxons required to seed the infant’s initial mucosal surfaces and guide normal neonatal immune development [45,46].

## 3. Microbiome Alterations in Gestational Diabetes Mellitus (GDM)

**Figure 1:**
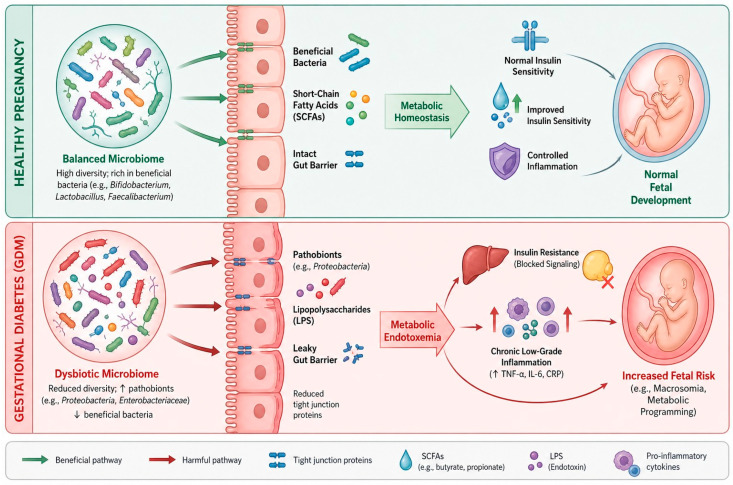
Gut microbiome–metabolic axis in healthy pregnancy versus gestational diabetes mellitus (GDM). The (**upper**) panel shows a balanced maternal microbiome that supports intestinal barrier integrity, short-chain fatty acid (SCFA) production, metabolic homeostasis, and normal fetal development. The (**lower**) panel illustrates GDM-associated dysbiosis, characterized by reduced diversity, increased pathobionts, and impaired gut barrier function, leading to lipopolysaccharide (LPS) translocation, chronic inflammation, and insulin resistance, ultimately increasing fetal metabolic risk. This model is conceptual; current evidence remains largely associative, and causal mechanisms are not fully established.

When a pregnancy is complicated by GDM, the natural adaptive shifts in gestation diverge into pathological dysbiosis [33]. Metagenomic profiling reveals a distinct taxonomic signature characterized by a severe loss of keys anti-inflammatory, butyrate-producing organisms. Taxons such as *Faecalibacterium prausnitzii*, *Roseburia intestinalis*, *Eubacterium rectale*, and *Coprococcus* undergo a profound reduction in relative abundance [48,49]. Conversely, there is a reciprocal, uncontrolled overgrowth of opportunistic, highly inflammatory Gram-negative pathobionts, specifically from the Enterobacteriaceae family (including *Escherichia coli* and *Klebsiella pneumoniae*), alongside an enrichment of *Ruminococcus gnavus* and *Bacteroides caccae* [49,50]. Furthermore, the highly beneficial, mucus-dwelling verrucomicrobiote *Akkermansia muciniphila*—which is required to maintain the structural integrity of the intestinal mucus layer by continuously degrading and stimulating mucus renewal—is often severely depleted in GDM cohorts [51]. This specific taxonomic shift strips the host of its primary protective physiological barrier, allowing pathobionts to come into direct structural contact with the underlying epithelial cell monolayer, severely accelerating the degradation of tight junction proteins and facilitating the uncontrolled systemic transit of inflammatory triggers [52] (Figure 1).

### 3.1. Taxonomic Shifts and Diversity Signatures in GDM Profiles

The onset of gestational diabetes mellitus (GDM) is fundamentally characterized by a significant disruption of the gut microbiota’s structural architecture, deviating sharply from the physiological adaptations observed in uncomplicated pregnancies. Recent high-throughput metagenomic sequencing studies demonstrate a pronounced reduction in microbial alpha diversity (within-sample richness and evenness) in women with GDM compared to healthy pregnant controls [53,54]. At the phylum level, this dysbiosis is manifested by a marked expansion of *Proteobacteria* and a high *Firmicutes*-to-*Bacteroidetes* ratio [54]. At the genus level, GDM cohorts exhibit a consistent enrichment of opportunistic pathobionts and pro-inflammatory taxons, such as *Klebsiella*, *Ruminococcus*, *Blautia*, and members of the *Enterobacteriaceae* family [53,54]. Conversely, there is a severe depletion of beneficial, health-promoting keystone genera, including *Faecalibacterium*, *Bifidobacterium*, *Roseburia*, and *Akkermansia* [54,55,56]. These profound taxonomic alterations do not merely represent a bystander effect of maternal hyperglycemia; rather, they induce a functional remodeling of the intestinal metagenome, shifting it away from homeostatic metabolic pathways toward pathways that promote metabolic endotoxemia and insulin resistance [56,57]. Collectively, these findings suggest that GDM is characterized by a reproducible microbial signature involving depletion of beneficial SCFA-producing and barrier-supporting taxa alongside enrichment of pro-inflammatory pathobionts. The principal microbial alterations consistently reported across GDM cohorts and their proposed biological significance are summarized in Table 1.

### 3.2. Metabolic Endotoxemia and the LPS/TLR4 Signaling Cascade

A primary molecular mechanism linking maternal gut dysbiosis to the pathogenesis of gestational diabetes mellitus (GDM) is the induction of low-grade systemic inflammation, driven by metabolic endotoxemia. In GDM patients, the enrichment of Gram-negative pathobionts—particularly within the Enterobacteriaceae family—leads to an overproduction of luminal lipopolysaccharides (LPSs) [53]. Concurrently, the loss of barrier-protective taxons compromises tight junction integrity, allowing for the paracellular translocation of LPS into the maternal portal circulation [53,54]. Once systemic, LPS binds to the toll-like receptor 4 (TLR4)/coreceptor MD-2 complex on the surface of maternal adipocytes, hepatocytes, and skeletal muscle cells [54]. This binding triggers the recruitment of MyD88, activating the inhibitor of nuclear factor-kB kinase β (IKKβ) and c-Jun N-terminal kinase (JNK) pathways [54]. These activated serine kinases induce the inhibitory serine phosphorylation (e.g., Ser307) of Insulin Receptor Substrate 1 (IRS-1), effectively blocking downstream phosphatidylinositol 3-kinase (PI3K)/Akt signaling and halting the translocation of glucose transporter 4 (GLUT4) to the cell membrane, which culminates in severe maternal insulin resistance [53,54].

### 3.3. Short-Chain Fatty Acid (SCFA) Deficiency and Incretin Dysregulation

Beyond endotoxemia, the metabolic failure characteristic of GDM is heavily exacerbated by a profound functional shift in the microbial metabolome, notably the depletion of short-chain fatty acids (SCFAs) such as acetate, propionate, and butyrate. Recent metagenomic profiling indicates a drastic reduction in key saccharolytic keystone species, including *Faecalibacterium prausnitzii* and *Roseburia intestinalis*, in women diagnosed with GDM [4]. The resulting deficit in luminal butyrate deprives colonocytes of their primary energy source, aggravating gut barrier leakiness [54,56]. Furthermore, a reduction in circulating acetate and propionate diminishes the activation of free fatty acid receptors 2 and 3 (FFAR2/3, formerly GPR43/41) expressed on intestinal enteroendocrine L-cells and pancreatic β-cells [56,57]. Under physiological conditions, FFAR2/3 signaling stimulates the secretion of glucagon-like peptide-1 (GLP-1) and peptide YY (PYY), which optimize glucose-dependent insulin secretion and peripheral insulin sensitivity [56]. The disruption of this symbiotic SCFA-FFAR2/3 axis in GDM leads to impaired incretin kinetics, inadequate compensatory pancreatic insulin output, and unmitigated hepatic gluconeogenesis, further accelerating gestational glucose intolerance [54,56].

### 3.4. Placental Translocation and Meconium Microbiome Signatures: From Controversy to Signaling

The concept of a distinct, functional placental microbiome has ignited one of the most intense debates in contemporary reproductive microbiology [58]. While early investigations suggested that the human placenta harbored a unique, low-abundance microbiome, subsequent rigorous studies utilizing advanced clean-room isolation protocols and exhaustive negative controls have overwhelmingly demonstrated that the healthy placenta is fundamentally sterile [57,59]. The bacterial sequencing signals identified in earlier studies are now widely acknowledged to stem from environmental contamination or extraction kit reagents (the “kitome”) [59].

Nevertheless, this lack of a live, resident placental microbiome does not mean the organ is isolated from maternal microbial signals [58]. In pregnancies complicated by GDM, the compromised maternal gut barrier allows the shedding of bacterial cell wall components, viable metabolic ligands, and non-viable bacterial DNA fragments to continuously escape into the maternal circulation [54]. These translocated microbial fragments are carried via the uterine circulation directly to the placenta, where they engage pattern recognition receptors, specifically TLR2 and TLR4 expressed on syncytiotrophoblasts [54,55]. This engagement triggers a localized inflammatory cascade characterized by the hyper-secretion of pro-inflammatory cytokines (TNF-α, IL-1β, and IL-6) into the fetal vascular circuit [54]. This metaflammation alters the expression and trafficking of critical nutrient transporters—such as sodium-coupled neutral amino acid transporters (SNATs) and glucose transporters (GLUT1)—fundamentally disrupting placental nutrient transfer dynamics and driving downstream fetal overgrowth [54,55].

Concurrently, this altered maternal–fetal metabolic environment is reflected within the earliest stages of fetal intestinal colonization, as evidenced by the evaluation of neonatal meconium [60]. Meconium samples collected from newborns exposed to maternal GDM in utero exhibit an aberrant, distinct microbial configuration long before the introduction of postnatal nutrition [60]. These meconium profiles feature an enriched abundance of opportunistic *Proteobacteria* and a corresponding depletion of protective, pioneering *Bifidobacterium* and *Lactobacillus* taxons [61,62,63,64]. This suggests that intrauterine exposure to maternal GDM derivatives alters the fetal amniotic fluid microenvironment, pre-conditioning the initial fetal gut lumen toward an inflammatory state prior to birth [60].

## 4. Mechanistic Pathways Linking the Microbiome to GDM Pathogenesis

The molecular landscape linking the maternal dysbiotic microbiome to the pathogenesis of GDM functions through four deeply integrated, overlapping pathophysiological axes, detailed below.

### 4.1. Microbial Metabolites and Insulin Resistance

The depletion of SCFA synthesis directly impairs the activation of G-protein coupled receptors GPR41 and GPR42 on enteroendocrine cells, downregulating the homeostatic release of the incretin hormone GLP-1 [10]. This lack of GLP-1 stimulation deprives the maternal pancreas of a key survival signal, accelerating beta-cell exhaustion under metabolic workloads. Concurrently, the dysbiotic microbiome increases the synthesis of trimethylamine (TMA) from dietary choline and carnitine, which is rapidly oxidized by hepatic flavin-containing monooxygenases into trimethylamine N-oxide (TMAO) [7]. High circulating TMAO directly activates the double-stranded RNA-dependent protein kinase (PKR), which intensifies hepatic gluconeogenesis while simultaneously blocking insulin signaling pathways in peripheral tissues. Furthermore, alterations in bacterial bile acid hydrolase (BAH) activity shift the circulating pool away from protective secondary bile acids, preventing the normal activation of TGR5 and FXR receptors, which are essential for coordinating systemic lipid clearance and insulin sensitivity [11].

### 4.2. Inflammation and Metabolic Endotoxemia

**Figure 2:**
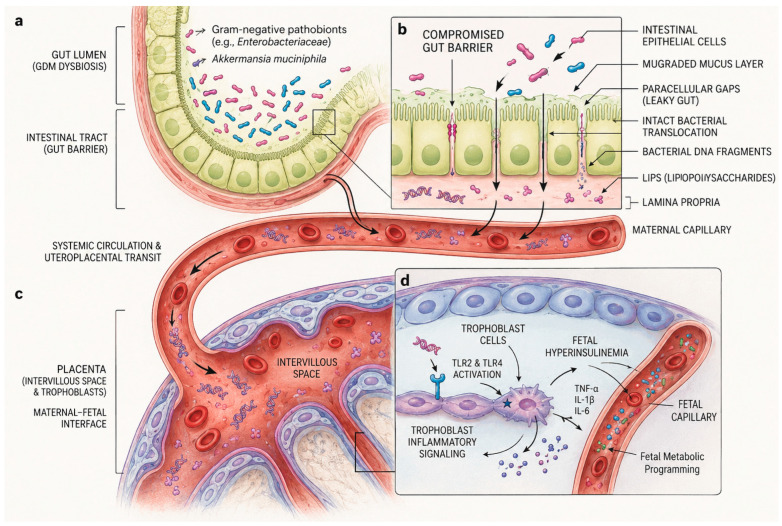
Mechanistic overview of maternal intestinal barrier dysfunction and uteroplacental microbial signaling in gestational diabetes mellitus (GDM). (**a**) Dysbiosis triggers the degradation of the mucosal shield and downregulates tight junctions, leading to paracellular gaps (leaky gut). (**b**) This facilitates the systemic translocation of intact pathobionts, bacterial DNA fragments, and lipopolysaccharides (LPSs) into maternal circulation (metabolic endotoxemia). (**c**) Upon reaching the intervillous space, these microbial derivatives activate trophoblast pattern recognition receptors (TLR2/TLR4). (**d**) initiating localized inflammatory cascades (TNF-alpha, IL-1-beta, IL-6) that perturb fetal metabolic programming and nutrient transport.

The overgrowth of Gram-negative *Enterobacteriaceae* causes a continuous accumulation of lipopolysaccharide (LPS) within the intestinal lumen [21] (Figure 2). Due to the breakdown of the mucosal barrier, this LPS enters the systemic circulation at pathogenic concentrations. Systemic LPS binds to the TLR4/CD14 receptor complex on circulating monocytes and tissue-resident macrophages [22]. This interaction initiates a intracellular signaling cascade via the MyD88 pathway, driving the nuclear translocation of NF-κB. This transcription factor upregulates the systemic expression of potent inflammatory cytokines, including TNF-alpha, IL-6, and MCP-1. These circulating cytokines induce the serine phosphorylation of IRS-1, uncoupling the insulin receptor from its downstream signaling components and effectively freezing insulin-mediated glucose disposal [24].

The expansion of Gram-negative members of the Enterobacteriaceae family has been consistently associated with increased luminal lipopolysaccharide (LPS) burden and features of metabolic endotoxemia in GDM cohorts. Concurrently, reduced abundance of barrier-supporting taxa, including *Akkermansia muciniphila* and butyrate-producing bacteria, may contribute to impaired intestinal barrier integrity and increased translocation of microbial products into the systemic circulation. Circulating LPS can interact with Toll-like receptor 4 (TLR4) and its co-receptor CD14 on monocytes, macrophages, adipocytes, and other metabolically active tissues, activating MyD88-dependent signaling pathways and promoting nuclear factor-κB (NF-κB) translocation. Subsequent upregulation of pro-inflammatory mediators, including tumor necrosis factor-α (TNF-α), interleukin-6 (IL-6), and monocyte chemoattractant protein-1 (MCP-1), has been implicated in the development of chronic low-grade inflammation characteristic of GDM. These inflammatory pathways may interfere with insulin signaling through inhibitory serine phosphorylation of insulin receptor substrate-1 (IRS-1), thereby contributing to impaired PI3K/Akt signaling, reduced glucose uptake, and progressive insulin resistance. However, it should be noted that most evidence supporting these mechanisms remains associative, and further studies are required to establish direct causal relationships in human pregnancy.

Strong support for a causal contribution of the microbiome comes from fecal microbiota transplantation experiments, in which first-trimester microbiota from women who later developed GDM transferred metabolic abnormalities to germ-free mice [29,41,62,63].

### 4.3. Gut Barrier Dysfunction

The structural failure of the intestinal barrier in GDM is driven by the active loss of mucus-protecting species like *Akkermansia muciniphila* and SCFA producers [51]. The resulting drop in luminal butyrate starves colonocytes of their primary energy source, inducing cellular stress and leading to the downregulation and internal degradation of the key tight junction proteins zonula occludens-1 (ZO-1), occludin, and claudins [22]. As these molecular seals degrade, the paracellular space between enterocytes widens significantly, creating a hyper-permeable “leaky” gut architecture. This structural failure permits the unmitigated, bulk translocation of intact viable pathobionts, bacterial cell wall fragments, and structural endotoxins directly into the mesenteric lymph nodes and portal vein, transforming a localized intestinal disruption into a systemic metabolic disaster [20].

### 4.4. Immune System Modulation

In a healthy pregnancy, the maternal immune system actively maintains a high threshold of mucosal tolerance, driven by the continuous generation of CD4+CD25+FoxP3+ regulatory T cells within the lamina propria, stimulated by microbial butyrate and specialized dendritic cells [45,65]. In GDM dysbiosis, the acute depletion of butyrate-producing taxons halts this protective differentiation pipeline. Instead, the persistent presentation of pathobiont antigens shifts the local immune profile toward a pro-inflammatory T helper 17 and Th1 phenotype. This immune balance failure leads to the hyper-secretion of IL-17 and IFN-gamma, which systematically destroys mucosal immune tolerance, amplifies local tissue destruction, and sustains a continuous state of systemic metaflammation that completely undermines maternal insulin sensitivity [15,16].

## 5. Maternal–Fetal Microbiome Interactions

The maternal–fetal microbial relationship is a key determinant of long-term offspring health. Beyond metabolite-mediated effects, the maternal microbiome serves as a primary source for early-life microbial colonization, shaping immune and metabolic programming. However, the extent and timing of direct microbial transfer remain debated, highlighting the need for clearer mechanistic evidence.

### 5.1. Vertical Microbial Transmission: The Mother as Primary Source

The establishment of the neonatal microbiome is not an arbitrary or stochastic postnatal event; it represents a tightly orchestrated, evolutionary process dominated by the direct vertical transmission of maternal microbial reservoirs [66]. The maternal gut, vagina, skin, and breast milk serve as the primary foundational sources for early infant colonization [42]. During a normal vaginal delivery, the neonate is exposed to the highly specialized maternal vaginal *Lactobacillus* shield, alongside trace amounts of maternal fecal taxons [6]. This initial biological exposure seeds the infant’s sterile mucosal surfaces with highly adaptive founder strains, most notably *Bifidobacterium* species (such as *Bifidobacterium longum* subsp. *infantis*), which possess specialized gene complexes dedicated to the highly efficient consumption of human milk oligosaccharides (HMOs) present in maternal colostrum and breast milk [42,67,68]. This vertical transmission pipeline provides the essential genetic and metabolic machinery required to educate the primitive neonatal immune system, guide the morphological development of the infant intestinal villi, and maintain early host-metabolic homeostasis [69].

### 5.2. Impact of GDM on the Neonatal Microbiota

In pregnancies complicated by GDM, this vertical transmission pipeline is profoundly distorted [70]. The altered, dysbiotic composition of the maternal gut and vaginal reservoirs means that the neonate is inoculated at birth with an aberrant, highly uncoupled microbial founder pool [70]. Longitudinal evaluation of infants born to GDM mothers demonstrates a marked delay in the normal colonization sequence; the acquisition of protective, beneficial *Bifidobacterium* and *Lactobacillus* strains is significantly depressed, while the neonatal gut features an immediate, persistent enrichment of opportunistic, pro-inflammatory pathobionts, including *Enterococcus*, *Staphylococcus*, and members of the Enterobacteriaceae family [71,72,73]. This early-life dysbiosis causes a functional deficiency in neonate SCFA production, depriving the infant intestinal epithelium of essential developmental signals and inducing a state of sub-clinical, low-grade systemic inflammation during critical windows of early development [65,74].

### 5.3. Long-Term Metabolic Programming

This early structural distortion of the neonatal founder microbiome under the influence of maternal GDM exerts profound, permanent effects via long-term metabolic programming [75]. The early-life loss of microbial diversity and the failure to establish robust SCFA production pathways disrupt the development of host energetic balance systems [76]. Metabolic programming pathways within the developing infant hypothalamus are highly sensitive to early microbial signals; a lack of healthy microbial metabolites during early life can downregulate the expression of pro-opiomelanocortin (POMC) satiety neurons while upregulating agouti-related peptide (AgRP) orexigenic pathways, permanently altering the child’s appetite regulation and energy expenditure systems [77]. Consequently, offspring exposed to maternal GDM and early postnatal dysbiosis exhibit a drastically elevated longitudinal risk of developing childhood obesity, early-onset metabolic syndrome, non-alcoholic fatty liver disease (NAFLD), and adolescent glucose intolerance, driving the intergenerational propagation of metabolic disease [4,35,67,78,79].

## 6. Microbiome-Based Biomarkers for Early GDM Detection

To bypass the diagnostic delays and clinical limitations inherent to the traditional 24–28 week oral glucose tolerance test, modern predictive medicine has pivoted toward identifying non-invasive, high-sensitivity biomarkers during the first trimester of gestation. By capturing subtle disruptions in the maternal intestinal ecosystem prior to the onset of overt systemic hyperglycemia, microbiome-derived taxonomic, metabolomic, and computational signatures offer an unprecedented window for early risk stratification and preventative intervention [53].

Recently, Yao et al. demonstrated that gut microbiota profiling during the first trimester (11–13 weeks of gestation) identified distinct microbial signatures associated with future GDM development, supporting the utility of microbiome-based early prediction strategies [78].

### 6.1. First-Trimester Taxonomic Signatures and Microbial Ratios

Prospective shotgun metagenomic and 16S rRNA amplicon sequencing cohorts have demonstrated that distinct alterations in gut microbial architecture are detectable long before clinical glucose intolerance manifests [80]. In early pregnancy, women destined to develop gestational diabetes mellitus display a significant contraction in intestinal alpha-diversity paired with sharp shifts in beta-diversity, indicating a prematurely destabilized baseline community structure characterized by a predictable binary divergence involving the progressive enrichment of pro-inflammatory pathobionts and a reciprocal collapse of highly specialized, health-promoting saccharolytic taxons [54,80].

At the taxonomic level, this first-trimester dysbiosis manifests as an overgrowth of specific genera, including *Blautia*, *Ruminococcus*, *Klebsiella*, *Eisenbergiella*, and *Fusobacterium*, which are directly implicated in mucosal barrier degradation, increased intestinal permeability, and the generation of local endotoxins [53,76]. Conversely, essential symbiotic genera responsible for maintaining intestinal immune tone, such as *Faecalibacterium*, *Akkermansia*, *Roseburia*, and *Bifidobacterium*, are significantly down-regulated [54,80].

Rather than tracking individual species, monitoring cooperative dynamics through specific microbial ratios yields far higher predictive accuracy. For instance, an elevated *Blautia*-to-*Faecalibacterium* ratio serves as a strong independent predictor of high fasting plasma glucose later in pregnancy by driving low-grade gut mucosal inflammation and compromising epithelial tight junctions [53]. Similarly, an elevated *Ruminococcus*-to-*Akkermansia* ratio reflects the active degradation of the protective colonic mucin layer by pathobionts and correlates heavily with elevated first-trimester HbA1c trajectories [76]. Furthermore, the enrichment of *Bacteroides vulgatus* is highly correlated with the biosynthesis and accumulation of branched-chain amino acids, driving early-stage peripheral insulin resistance, while a severe depletion of *Akkermansia muciniphila* thins the mucosal barrier and accelerates metabolic endotoxemia, signaling impending metabolic failure [54,80].

### 6.2. Functional Metabolomic Biomarkers: Fecal and Serum Profiles

While taxonomic profiling maps the structural composition of the maternal gut, functional metabolomics deciphers its biochemical output, revealing that the functional decline of the maternal gut ecosystem alters both the local luminal environment and the systemic maternal circulation during early gestation. Metagenomic functional pathway analysis reveals a profound down-regulation of microbial genes dedicated to carbohydrate fermentation and short-chain fatty acid biosynthesis during the first trimester [54]. Consequently, a marked contraction of fecal butyrate, acetate, and propionate serves as a primary functional biomarker triad [56]. A deficit in colonic butyrate deprives colonocytes of their vital energetic substrate, leading to subclinical intestinal barrier leakiness that allows for the systemic translocation of lipopolysaccharides, rendering elevated maternal serum lipopolysaccharide levels a highly sensitive, indirect biomarker of early-stage gut-derived metabolic endotoxemia [53,56].

Parallel to the short-chain fatty acid deficit, an overgrowth of specific amino acid-fermenting pathobionts leads to an upregulation of biosynthetic pathways for branched-chain amino acids and aromatic amino acids [54]. Elevated levels of leucine, isoleucine, valine, phenylalanine, and tyrosine in maternal serum during early pregnancy impair skeletal muscle glucose uptake by chronically overactivating the mTORC1 pathway, causing early insulin receptor desensitization [54,81]. Concurrently, an enrichment of choline-degrading bacteria drives an early rise in circulating trimethylamine N-oxide, a well-documented pro-inflammatory metabolite that exacerbates vascular inflammation and adipose tissue macrophage infiltration [81]. This is further compounded by an altered serum ratio of primary to secondary bile acids resulting from a distinct loss of bacteria possessing bile salt hydrolase activity [82]. This functional loss disrupts the normal conversion of primary bile acids into secondary bile acids, and the resulting primary-skewed bile acid pool diminishes the systemic activation of the Takeda G-protein-coupled receptor 5 and the farnesoid X receptor on intestinal L-cells, serving as an exceptionally sensitive predictor of blunted glucagon-like peptide-1 kinetics and inadequate late-gestational insulin secretion [56,82].

### 6.3. Advanced Multi-Omic Integration and Machine Learning Classifiers

The translation of these complex microbial and metabolomic datasets into clinically actionable tools relies on integrating multi-omic vectors with traditional clinical parameters using machine learning algorithms. Recent multi-center trials have validated integrated predictive frameworks that synthesize first-trimester taxonomic variables, microbial metabolomic inputs, and traditional clinical covariates such as maternal age, pre-pregnancy body mass index, ethnicity, and early fasting plasma glucose [80,83].

When these multi-omic vectors are processed through advanced classifiers like random forest, gradient boosting, or support vector machines, they routinely achieve an area under the receiver operating characteristic curve ranging from 0.82 to 0.89 [31,80,83]. These integrated models vastly outperform traditional clinical screening risk grids, which typically yield an area under the curve of only 0.65 to 0.70. By shifting the diagnostic timeline from the late second trimester to the early first trimester, these machine-learning-driven multi-omic frameworks allow clinical teams to stratify maternal risk dynamically, creating a vital window of opportunity to implement targeted, microbiome-focused preventive measures months before the onset of overt clinical hyperglycemia [54,81].

Several microbial and microbiome-derived metabolites have emerged as promising candidates for first-trimester prediction of GDM. These biomarkers reflect both taxonomic alterations and functional metabolic changes and may improve risk stratification when combined with clinical parameters. The most consistently reported candidate biomarkers are summarized in Table 2.

The integration of microbial taxa and microbiome-derived metabolites may provide greater predictive power than individual biomarkers alone. Recent studies suggest that combining microbial composition, metabolomic signatures, and clinical variables through machine-learning algorithms may improve first-trimester risk stratification and facilitate personalized preventive interventions.

### 6.4. Machine Learning and Artificial Intelligence for Early GDM Prediction

The increasing availability of microbiome sequencing data has stimulated the application of artificial intelligence (AI) and machine learning (ML) approaches for the early prediction of gestational diabetes mellitus. Traditional clinical risk factors, including maternal age, BMI, family history, and fasting glucose, provide only moderate predictive accuracy during early pregnancy. In contrast, ML algorithms can integrate high-dimensional microbiome, metabolomic, and clinical datasets to identify complex non-linear patterns associated with future GDM development.

Random Forest models have been the most frequently applied approach due to their robustness when handling microbiome compositional data. Several studies have demonstrated that the inclusion of microbial taxa such as *Blautia*, *Faecalibacterium*, *Akkermansia*, and members of the Enterobacteriaceae family significantly improves predictive performance compared with clinical variables alone. More recently, gradient boosting algorithms such as XGBoost and LightGBM have achieved higher classification accuracy by integrating microbiome profiles with serum metabolites including branched-chain amino acids (BCAAs), trimethylamine-N-oxide (TMAO), and short-chain fatty acids.

Popova et al. developed a machine-learning framework integrating continuous glucose monitoring, dietary records, clinical variables, and gut microbiome profiles from 105 pregnant women. Inclusion of microbiome-derived features improved prediction of postprandial glycemic responses compared with carbohydrate-based models alone, highlighting the potential of microbiome-assisted precision nutrition in GDM management [36].

Recent metagenomic studies incorporating machine-learning approaches have demonstrated promising predictive performance for GDM classification based on microbial signatures, highlighting the growing potential of microbiome-driven precision diagnostics.

Emerging multi-omics frameworks combine metagenomics, metabolomics, transcriptomics, and clinical metadata to generate personalized risk scores. Such approaches may facilitate first-trimester risk stratification and support precision medicine strategies for individualized prevention. Nevertheless, current AI models remain limited by small cohort sizes, lack of external validation, population-specific microbial signatures, and methodological heterogeneity. Large prospective multicenter studies will be required before AI-assisted microbiome diagnostics can be translated into routine obstetric practice [90].

## 7. Microbiome-Based Interventions

**Figure 3:**
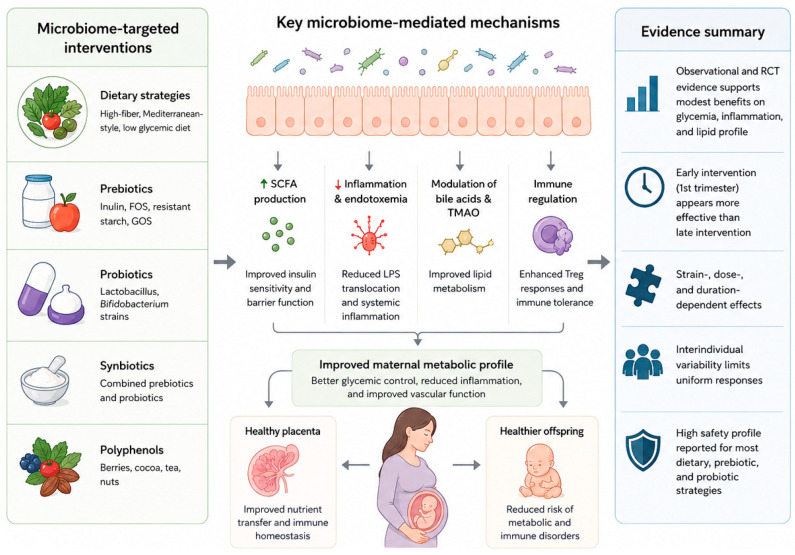
Microbiome-targeted interventions in GDM can be done using dietary and microbiome-based interventions—including high-fiber and Mediterranean diets, prebiotics, probiotics, synbiotics, and polyphenols—and their proposed mechanisms of action. These strategies modulate gut microbial composition and function, enhancing short-chain fatty acid (SCFA) production, improving gut barrier integrity, reducing endotoxemia, and regulating immune and metabolic pathways, ultimately contributing to improved maternal metabolic profile and potential benefits for placental function and offspring health.

Restabilizing the maternal microbiome during early pregnancy represents a powerful, highly scalable therapeutic opportunity to mitigate metaflammation and intercept the development of GDM (Figure 3). These interventions are deployed across three primary pillars.

### 7.1. Dietary and Nutritional Interventions

The foundational strategy involves modulating the maternal diet to enrich the internal luminal environment with complex carbohydrate substrates [91]. Transitioning pregnant patients to a strict Mediterranean dietary pattern—characterized by high consumption of non-digestible soluble fibers, polyphenols, and monounsaturated fatty acids—provides an abundance of metabolic prebiotics [92]. Saccharolytic bacteria utilize these substrates to rapidly upregulate SCFA synthesis, which actively reinforces the intestinal tight junction complexes, lowers luminal pH to suppress pathobionts, and significantly reduces systemic endotoxemia [93,94]. Furthermore, dietary polyphenols act as natural antimicrobial agents against Gram-negative pathobionts while selectively supporting the proliferation of beneficial *Bifidobacterium* and *Faecalibacterium* strains [95,96].

### 7.2. Probiotics and Synbiotics

Clinical deployment of targeted probiotic formulations during early gestation has demonstrated significant efficacy in optimizing maternal glucose homeostasis [97]. Randomized, double-blind, placebo-controlled clinical trials have shown that multi-strain probiotic interventions—combining highly characterized strains such as *Lactobacillus rhamnosus* GG, *Bifidobacterium animalis* subsp. *lactis*, and *Lactobacillus acidophilus*—significantly lower maternal fasting plasma glucose, reduce homeostatic model assessment for insulin resistance (HOMA-IR) values, and suppress circulating pro-inflammatory cytokine profiles [98,99]. When these live probiotic strains are structurally paired with matching prebiotic substrates—a configuration known as a synbiotic—the survival, luminal colonization efficiency, and metabolic output of the therapeutic strains are maximized, offering enhanced clinical protection [100].

### 7.3. Prebiotics

Utilizing isolated prebiotic formulations, such as inulin, fructooligosaccharides (FOSs), and galactooligosaccharides (GOSs), provides a highly targeted method to modulate the endogenous maternal microbiota [101]. Prebiotics pass completely unabsorbed through the upper maternal gastrointestinal tract, arriving intact within the colon where they act as exclusive, high-affinity substrates for native, beneficial bifidobacteria and specific butyrate producers [102]. Regular prebiotic intake during early pregnancy drives a sustained enrichment of these protective taxons, leading to a continuous downregulation of host systemic metaflammation and significantly lowering postprandial glucose excursions in high-risk pregnant cohorts [103].

## 8. Methodological Challenges in Microbiome and GDM Studies

Despite the immense clinical promise of microbiome-based diagnostics and therapeutics, the field faces substantial methodological hurdles that limit the direct translation of research findings into standardized clinical guidelines [103]. A primary obstacle is extensive methodological heterogeneity, as the cross-study comparison of gestational diabetes mellitus (GDM) microbiome datasets is severely compromised by widespread variations across experimental pipelines [103]. Discrepancies routinely emerge at every operational stage, including the choice of fecal collection devices, storage buffers, transport temperatures, and DNA extraction chemistries [104]. For instance, different commercial DNA extraction kits exhibit highly disparate lysis efficiencies when processing tough, peptidoglycan-rich Gram-positive cell walls versus fragile Gram-negative outer membranes, introducing profound artificial skewing into the final taxonomic ratios and alpha-diversity indices reported across the literature [105].

**Figure 4:**
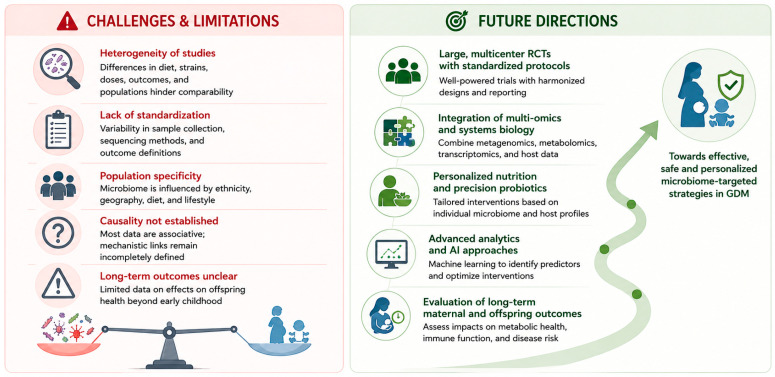
Challenges and future directions in GDM.

This technical bias is further compounded by limitations in sequencing depth and resolution, given that a large proportion of the historical GDM microbiome literature relies heavily on 16S rRNA gene amplicon sequencing [96]. While 16S sequencing provides a high-throughput, cost-effective method to map broad phylum- and genus-level taxonomic shifts by targeting hypervariable regions, it fundamentally lacks the structural resolution required to identify individual bacterial species or distinct, strain-level functional variations that dictate metabolic phenotypes [96]. Shifting the clinical research paradigm toward deep, shotgun metagenomic sequencing effectively resolves this taxonomic limitation by capturing the entire genomic repertoire; however, it introduces significant computational challenges, high financial costs, and substantial batch amplification effects that necessitate advanced, non-standardized bioinformatic correction and multi-omic integration pipelines [106,107] (Figure 4).

Beyond laboratory instrumentation artifacts, human gestational cohorts are inherently and highly susceptible to an array of confounding variables that are frequently under-controlled or omitted in clinical study designs [108].

Interpretation of microbiome-based biomarkers in GDM remains challenging due to substantial interindividual variability and multiple confounding factors. Maternal BMI, dietary patterns, ethnicity, socioeconomic status, antibiotic exposure, gestational weight gain, delivery mode, and pre-pregnancy metabolic health significantly influence microbial composition and metabolic profiles. These variables may contribute to inconsistencies between studies and currently limit the reproducibility and generalizability of microbiome-derived biomarkers across different populations [108]. Failing to rigorously match experimental cohorts or computationally control for these multi-layered biological variables through robust multivariate statistics frequently leads to false-positive taxonomic associations, creating ephemeral biomarkers that limit the cross-population validity and generalizability of discovered diagnostic signatures [108].

Finally, these limitations reach a critical threshold during the investigation of low-biomass contamination, where examining highly protected niches—such as amniotic fluid, placental tissues, or early neonatal meconium—carries a profound risk of environmental and technical noise [109]. In these specialized, low-biomass environments, the absolute quantity of authentic target microbial DNA is frequently equal to or lower than the background trace DNA natively present within laboratory extraction reagents, PCR master-mixes, and plastic collection tubes, a phenomenon widely documented as the “kitome” [109]. Consequently, without the mandatory inclusion of exhaustive negative extraction controls, clean-room isolation facilities, and sophisticated computational decontamination algorithms capable of filtering background signals, trace environmental contamination can be easily misidentified as a novel, live intrauterine microbial ecosystem, misguiding mechanistic models of fetal programming and pathogenesis [109].

## 9. Future Perspectives: Toward Precision Medicine in GDM

The future of managing gestational diabetes mellitus lies in moving away from reactive, one-size-fits-all clinical protocols toward highly integrated, multi-omic precision medicine (Figure 4). By combining longitudinal maternal microbiome profiling with deep serum metabolomics, transcriptomics, and maternal genetic typing, the next generation of prenatal care will leverage advanced artificial intelligence models to generate dynamic, personalized metabolic risk scores during the earliest days of the first trimester [106,107].

Emerging microbiome-informed frameworks propose that GDM should be viewed not solely as a disorder of glucose regulation but also as a condition influenced by host–microbiome–metabolome interactions, opening new opportunities for precision prevention and treatment [110].

To move beyond simple correlation, future research must prioritize mechanistic validation using highly humanized germ-free animal designs and cutting-edge organ-on-a-chip technologies to establish definitive causality [108]. Clinical intervention pipelines will transition from generic, off-the-shelf probiotic formulations toward targeted, next-generation live biotherapeutic products (LBPs) designed to repair specific functional metabolic deficiencies identified within an individual patient’s metagenomic profile [7]. Furthermore, large-scale, international multicenter longitudinal clinical trials are urgently required to validate these multi-omic predictive algorithms across highly diverse racial, ethnic, and socio-economic populations, establishing standardized global guidelines for early intervention [109]. Ultimately, mastering the therapeutic modulation of the maternal microbiome represents a powerful clinical opportunity to protect immediate maternal–fetal health and break the intergenerational cycle of metabolic disease on a global scale [111].

Recent clinical evidence highlights that targeted microbiota modulation can significantly alleviate systemic inflammation, liver stiffness, steatosis, and overall gut–liver axis dysfunction [111]. These findings open promising horizons for future microbiome-based therapeutic designs in metabolic diseases. However, it must be explicitly emphasized that aggressive interventions such as Fecal Microbiota Transplantation (FMT) remain entirely experimental at this stage. Recent clinical evidence further supports the concept that microbiota modulation may influence systemic metabolic and inflammatory pathways beyond glycemic regulation. A recent study demonstrated that fecal microbiota transplantation (FMT) was associated with improvements in systemic inflammation, hepatic steatosis, liver stiffness, and gut–liver axis dysfunction, highlighting the broader therapeutic potential of microbiome-targeted interventions in metabolic disease states [111]. These findings reinforce the concept that microbial modulation may influence host metabolic homeostasis through interconnected immune and endocrine pathways. However, despite these promising observations, FMT remains an experimental approach, particularly in pregnancy. Significant concerns persist regarding donor selection, pathogen transmission, long-term maternal–fetal safety, and the absence of robust clinical trials evaluating FMT in pregnant populations. Therefore, such interventions should currently be interpreted cautiously and should not be extrapolated to gestational diabetes management without dedicated safety and efficacy studies. Due to the lack of robust safety profiling and potential risks to both maternal and fetal health, FMT data from general metabolic cohorts cannot be extrapolated directly to pregnant populations without dedicated, strictly controlled clinical trials [111].

## 10. Conclusions

GDM should no longer be viewed solely as a disorder of glucose metabolism, but rather as a multifactorial condition arising at the interface of host metabolism, immune regulation, and the maternal microbiome. Accumulating evidence indicates that microbial dysbiosis is not merely a secondary consequence of metabolic imbalance but may act as an upstream driver of insulin resistance through mechanisms such as metabolic endotoxemia, immune activation, and disruption of intestinal barrier integrity. This perspective carries important clinical implications, as it opens the possibility of shifting from late, reactive diagnosis toward early, mechanism-based intervention. Current screening strategies, largely dependent on glycemic measurements in the second trimester, fail to capture early pathophysiological changes; therefore, the integration of microbiome-derived biomarkers—including taxonomic signatures such as the depletion of *Akkermansia* and *Bifidobacterium*, as well as functional metabolites like short-chain fatty acids and TMAO—may enable first-trimester risk stratification and more timely preventive strategies. In this context, modulation of the microbiome through personalized nutrition, prebiotics, probiotics, and emerging postbiotic approaches represents a promising therapeutic avenue, although robust clinical validation is still required before routine implementation. Translating these insights into clinical practice will require a paradigm shift toward early screening models that incorporate microbiome and metabolomic data, the development of individualized dietary recommendations based on microbial profiles, and the recognition of the microbiome as both a biomarker and a therapeutic target within multidisciplinary care frameworks. At the same time, several critical research gaps must be addressed, including the need for longitudinal human studies to establish causality, standardization of microbiome methodologies to improve reproducibility, deeper mechanistic investigation of the gut–placenta axis, and the development of integrative multi-omic predictive models that combine metagenomics, metabolomics, and host genetics. Furthermore, identifying distinct GDM endotypes will be essential for advancing precision medicine approaches and tailoring interventions to individual patients. Ultimately, redefining GDM through the lens of the microbiome provides a unique opportunity to move beyond symptomatic management and toward prevention, with the potential to disrupt the intergenerational transmission of metabolic disease and establish the maternal microbiome as a central target in future obstetric care.

## Figures and Tables

**Table 1:** Major microbiome alterations reported in gestational diabetes mellitus (GDM); ↓ decreased abundance, ↑ increased abundance.

Taxon	Direction	Proposed Biological Significance	Key References
*Akkermansia muciniphila*	↓	Maintenance of mucus layer integrity and gut barrier function	[51,55,56]
*Faecalibacterium prausnitzii*	↓	Butyrate production and anti-inflammatory effects	[48,49,55]
*Bifidobacterium* spp.	↓	Immune regulation and carbohydrate metabolism	[55,56]
Enterobacteriaceae	↑	Lipopolysaccharide production and metabolic endotoxemia	[49,53,54]
*Blautia*	↑	Associated with impaired glucose metabolism and insulin resistance	[53,55]
*Ruminococcus*	↑	Mucin degradation and intestinal permeability	[50,53]
*Klebsiella*	↑	Pro-inflammatory activity and endotoxin production	[53,55]
*Roseburia*	↓	Short-chain fatty acid production and metabolic homeostasis	[48,55,56]

**Table 2:** Candidate microbiome-derived biomarkers for early prediction of gestational diabetes mellitus.

Biomarker	Sample	Trimester	Predictive Significance	Key References
*Akkermansia muciniphila* abundance	Stool	T1	Reduced abundance associated with impaired gut barrier function and future GDM risk	[29,63]
*Blautia/Faecalibacterium* ratio	Stool	T1	Reflects inflammatory dysbiosis and insulin resistance	[33,84]
SCFAs (acetate, propionate, butyrate)	Stool/Serum	T1	Reduced concentrations associated with altered glucose metabolism	[63,85]
TMAO	Serum	T1	Elevated levels predict insulin resistance and GDM development	[86,87]
Branched-chain amino acids (BCAAs)	Serum	T1	Increased levels associated with impaired glucose tolerance and future GDM	[88,89]

## Data Availability

No new data were created or analyzed in this study. Data sharing is not applicable to this article.

## Abbreviations

GDM	Gestational Diabetes Mellitus
SCFAs	Short-Chain Fatty Acids
LPS	Lipopolysaccharides
OGTT	Oral Glucose Tolerance Test
T2DM	Type 2 Diabetes Mellitus
GLP-1	Glucagon-Like Peptide-1
TNF-α	Tumor Necrosis Factor-alpha
IL-6	Interleukin-6
ZO-1	Zonula Occludens-1
TLR4	Toll-Like Receptor 4
IRS-1	Insulin Receptor Substrate 1
IADPSG	International Association of Diabetes and Pregnancy Study Groups
